# Uncertainty in measurements of the photorespiratory CO_2_ compensation point and its impact on models of leaf photosynthesis

**DOI:** 10.1007/s11120-017-0369-8

**Published:** 2017-03-28

**Authors:** Berkley J. Walker, Douglas J. Orr, Elizabete Carmo-Silva, Martin A. J. Parry, Carl J. Bernacchi, Donald R. Ort

**Affiliations:** 1 Global Change and Photosynthesis Research Unit, United State Department of Agriculture/Agricultural Research Services, University of Illinois, 1206 W Gregory Dr., Urbana, IL 61801 USA; 2 Carl R. Woese Institute for Genomic Biology, University of Illinois, Urbana, IL 61801 USA; 3 Biochemistry of Plants, Heinrich-Heine University, Düsseldorf, Germany; 4 Lancaster Environment Centre, Lancaster University, Lancaster, LA1 4YQ UK; 5 Plant Biology and Crop Science, Rothamsted Research, Harpenden, AL5 2JQ UK; 6 Department of Plant Biology, University of Illinois, Urbana, IL 61801 USA

**Keywords:** Rubisco, Photorespiration, Temperature response, Modeling photosynthesis

## Abstract

Rates of carbon dioxide assimilation through photosynthesis are readily modeled using the Farquhar, von Caemmerer, and Berry (FvCB) model based on the biochemistry of the initial Rubisco-catalyzed reaction of net C_3_ photosynthesis. As models of CO_2_ assimilation rate are used more broadly for simulating photosynthesis among species and across scales, it is increasingly important that their temperature dependencies are accurately parameterized. A vital component of the FvCB model, the photorespiratory CO_2_ compensation point (Γ_*_), combines the biochemistry of Rubisco with the stoichiometry of photorespiratory release of CO_2_. This report details a comparison of the temperature response of Γ_*_ measured using different techniques in three important model and crop species (*Nicotiana tabacum, Triticum aestivum*, and *Glycine max*). We determined that the different Γ_*_ determination methods produce different temperature responses in the same species that are large enough to impact higher-scale leaf models of CO_2_ assimilation rate. These differences are largest in *N. tabacum* and could be the result of temperature-dependent increases in the amount of CO_2_ lost from photorespiration per Rubisco oxygenation reaction.

**Electronic supplementary material:**

The online version of this article (doi:10.1007/s11120-017-0369-8) contains supplementary material, which is available to authorized users.

## Introduction

Biochemical models of leaf photosynthesis are increasingly important as we develop more sophisticated simulations of plant carbon budgets and search for new strategies to improve crop productivity (Zhu et al. 2008; Dufresne et al. 2013; Long et al. 2015; Kromdijk and Long 2016). The widely adopted biochemical model of leaf photosynthesis of Farquhar, von Caemmerer, and Berry (FvCB) has proven invaluable since its development over 35 years ago and continues to be employed to represent photosynthesis from the cell to global scale (Farquhar et al. 1980; von Caemmerer and Farquhar 1981; von Caemmerer 2000). This model is characterized by its elegant combination of Rubisco kinetics with the physiology of photosynthesis and photorespiration to simulate net CO_2_ assimilation rate in response to CO_2_ partial pressures, making it useful both for predicting rates of carbon uptake as well as probing plant physiology and metabolism.

The photorespiratory CO_2_ compensation point (Γ_*_) is a critical parameter of the FvCB model. Γ_*_ integrates Rubisco specificity for reaction with CO_2_ relative to O_2_ (*S*_C/O_) with the stoichiometry of CO_2_ release per Rubisco oxygenation (*α*) to quantify photorespiratory CO_2_ loss to net CO_2_ assimilation rate. Γ_*_ is measured in three main ways, which can be understood in light of the following equation set:

1 $${{{\varGamma}}_*} = \frac{{\alpha O}}{{{S_{{\text{C/O}}}}}} = \frac{{\alpha {C_{\text{c}}}{v_{\text{o}}}}}{{{v_{\text{c}}}}},$$

where *O*, *C*_c_, *v*_o_, and *v*_c_ represent the oxygen partial pressure, chloroplastic CO_2_ partial pressure, rate of Rubisco oxygenation, and the rate of Rubisco carboxylation, respectively (Ruuska et al. 2000; von Caemmerer 2000; Walker and Cousins 2013). Γ_*_ has been measured in vivo using the common intersection method by measuring CO_2_ exchange under various CO_2_ partial pressures and irradiances and requires no assumed *α* value (Laisk 1977; Brooks and Farquhar 1985). Γ_*_ can also be calculated from in vitro determinations of *S*_C/O_ values as a function of O_2_ partial pressure, assuming that *α* equals 0.5 as predicted from the commonly accepted biochemistry of photorespiration (von Caemmerer 2000; Hermida-Carrera et al. 2016). Γ_*_ can also be determined from net oxygen fluxes in and out of the leaf using online mass spectroscopy (Badger ; Ruuska et al. 2000; Walker and Cousins 2013). The oxygen exchange method de^1985^termines the *C*_c_ *v*_o_/*v*_c_ ratio and *α* is assumed to equal 0.5.

Recently, there has been a growing interest in parameterizing the FvCB model with species-specific temperature responses of Rubisco to better represent photosynthesis and identify optimal Rubisco kinetics for given environments (e.g., Zhu et al. 2004; Walker et al. 2013; Hermida-Carrera et al. 2016; Orr et al. 2016). These efforts predominantly employ calculations based on in vitro *S*_C/O_ values due to the higher throughput of the technique, but it is not known how well in vitro *S*_C/O_ values compare to in vivo approaches like CO_2_ exchange and O_2_ exchange.

A recent compilation of Rubisco kinetics explored the differences among various in vitro and in vivo values and their impact on leaf-level modeling of net CO_2_ assimilation rate (Galmés et al. 2016). This meta-analysis supported the past work exploring the variability of Rubisco temperature responses from species adapted to different environments and contained an in-depth re-calculation of the past in vitro values with standard assumptions of ionic strength and gas solubilities. It also explored the differences between in vitro and in vivo methods and their sensitivity to modeled temperature responses, suggesting that both methods are useful for understanding and modeling the impact of Rubisco catalytic properties on photosynthesis based on their independent assumptions. The in vivo datasets analyzed in this paper were limited to those examining CO_2_ flux (Harley et al. 1985; Bernacchi et al. 2001; Walker et al. 2013), leading us to wonder what additional insights could be determined from methods based on O_2_ exchange, specifically in regards to measurements of Γ_*_ (Bernacchi et al. 2002). Additionally, Galmés et al. (2016) focused on methodological explanations of differences between the measured Rubisco kinetics, leading us to further question if there were any additional physiological explanations for the differences.

Differences among methods could indicate errors of some underlying physiological assumptions of the techniques. For example, it has been shown that Γ_*_ determined by CO_2_ exchange increases more with temperature than Γ_*_ determined by O_2_ exchange in *Arabidopsis thaliana* (Walker and Cousins 2013). Similar differences were observed between Γ_*_ determined in more extensive temperature response measurements in *Nicotiana tabacum* (Bernacchi et al. 2001, 2002, Fig. 1). Walker and Cousins (2013) suggested that the increased temperature response of Γ_*_ determined by CO_2_ exchange could be the result of an increase in *α* with temperature, but this hypotheses could not be confirmed due to other possible explanations inherent to determining O_2_ exchange using online mass spectroscopy (Walker and Cousins 2013). Like the O_2_ exchange method, Γ_*_ determined from in vitro *S*_C/O_ is also sensitive to the assumptions of *α*, so the secondary goal of this report was to observe if there were differences between Γ_*_ determined by CO_2_ exchange and in vitro *S*_C/O_ consistent with an increase in *α*. Measuring Γ_*_ from CO_2_ exchange involves the additional complication of converting the CO_2_ photocompensation point as measured from the intercellular CO_2_ partial pressure $(C_{\text{i}*})$ to chloroplastic partial pressures using values of day respiration (*R*_d_) and mesophyll conductance (*g*_m_, see “Materials and methods” section). This conversion relies on the assumed values of *g*_m_ and could also play a role in explaining the differences in Γ_*_ as determined using various techniques.

**Fig. 1:**
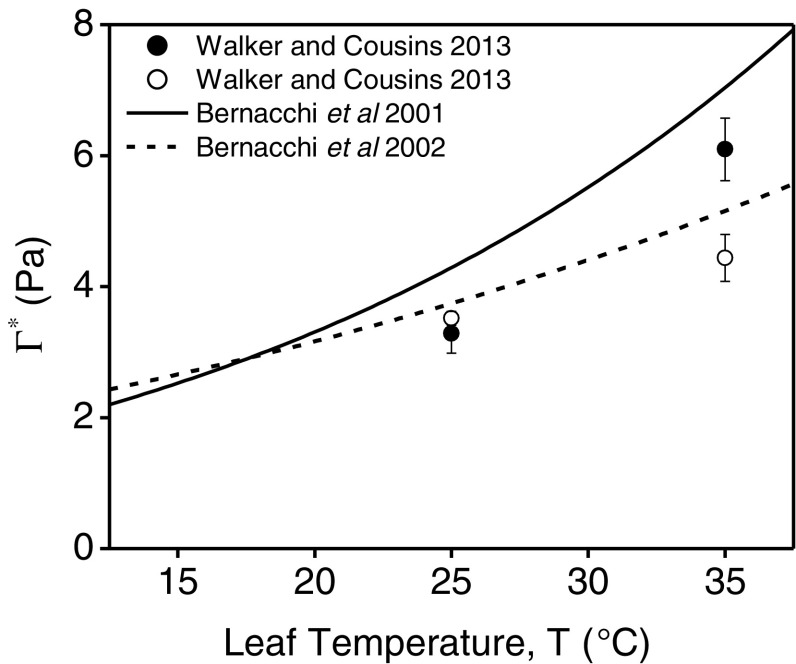
Comparison of the temperature response of the photorespiratory CO_2_ compensation point (Γ_*_) measured from CO_2_ gas exchange using the common intersection method (*closed circles* and Bernacchi et al. 2001) and from O_2_ gas exchange (*open circles* and Bernacchi et al. 2002)

In this report, the temperature response of Γ_*_ was measured using CO_2_ exchange and in vitro *S*_C/O_ in a C_3_ model species (*N. tabacum*) and two major C_3_ crop species (*Triticum aestivum* and *Glycine max*) to understand how comparable these methods are for use in simulating carbon assimilation at the leaf and canopy scale. This report demonstrates that there are differences between Γ_*_ determined by CO_2_ exchange and in vitro *S*_C/O_ that increase with temperature. These differences are most evident in *N. tabacum* and clearly present to a lesser extent in *T. aestivum* and *G. max*. The differences in the Γ_*_ temperature response, particularly for *N. tabacum*, are large enough to impact the output of leaf and canopy models of carbon assimilation parameterized with field data. Furthermore, differences in the Γ_*_ temperature response determined using CO_2_ exchange, in vitro *S*_C/O_, and O_2_ exchange in *N. tabacum* are consistent with an increase in *α* with temperature. These findings have important implications to how the FvCB model is parameterized and raise questions concerning one of its underlying assumptions that the stoichiometry of CO_2_ release per Rubisco oxygenation (*α*) is always 0.5.

## Results

The common intersection measurements used to derive the slope–intercept regression values of $C_{\text{i}*}$ and *R*_d_ produced consistent intersection points for a given temperature and species and were highly reproducible (Supplemental 1a–c). The different light intensities where the CO_2_ response of assimilation (*A*–*C*_i_) was measured produced an even distribution of slopes and intercepts for each temperature and species, with the exception of 15 and 20 °C in *G. Max* (Supplemental 1b). Additionally, due to the low values of CO_2_ partial pressures used during the measurement, the linear regressions of each *A*–*C*_i_ curve used *A*–*C*_i_ data taken exclusively from the most linear region of the *A*–*C*_i_ curve. The common intersection point of these linear regressions showed typical variations for each temperature and species, but there was no consistent trend in how well the lines intersected as a function of temperature. When the slopes and the y-intercepts of these individual lines were used to determine $C_{\text{i}*}$ using slope–intercept regression (Walker and Ort 2015; Walker et al. 2016a), there was no clear pattern in the residuals of the slope values between the linear regression and the measured values (Supplemental 2). This lack of pattern in the residual plots indicates that the slope–intercept regression was not measurably non-linear, indicating that a single *g*_m_ term is adequate to describe CO_2_ transfer to and from the chloroplast (Tholen and Zhu 2011; Tholen et al. 2012; Walker and Ort 2015; Walker et al. 2016a).

**Fig. 2:**
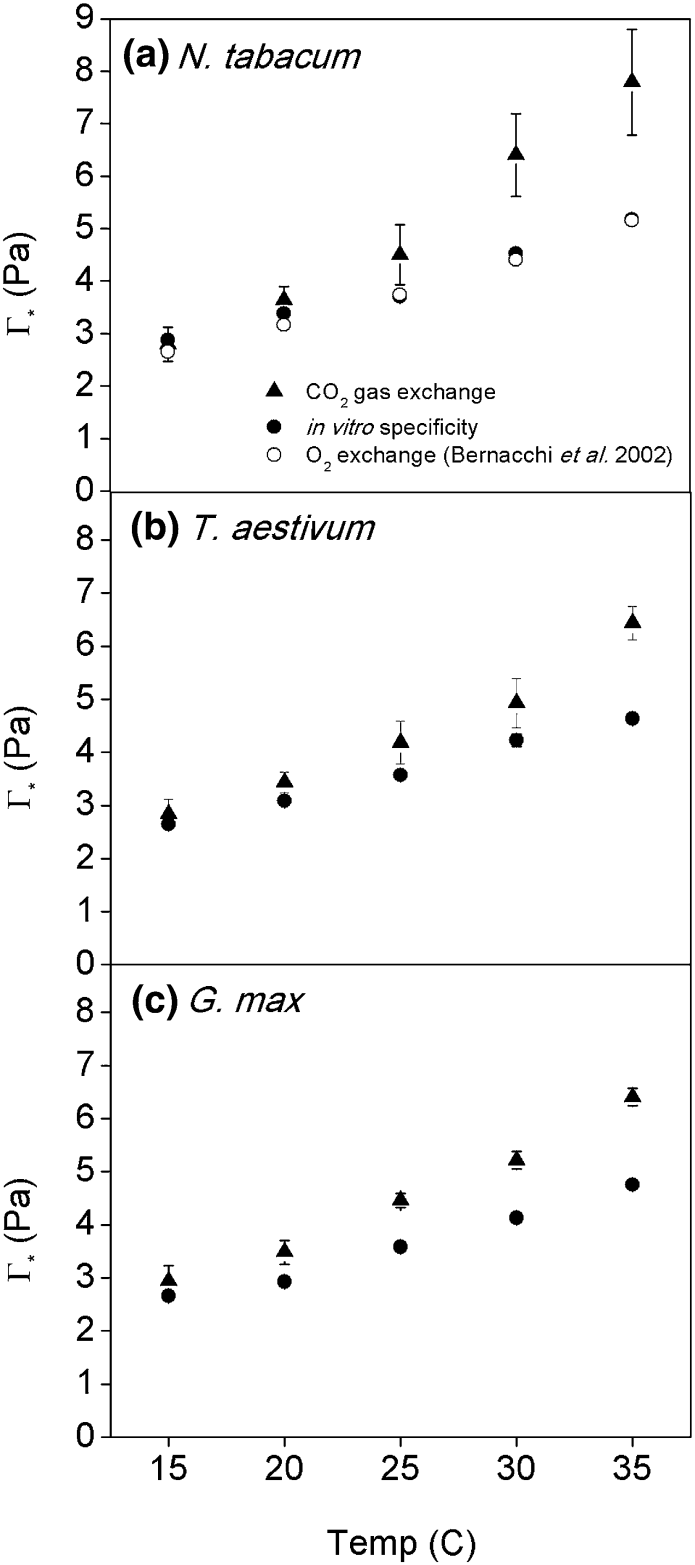
Temperature response of the photorespiratory CO_2_ compensation point (Γ_*_) measured from CO_2_ gas exchange using the common intersection method (*solid triangle*), calculated from Rubisco specificity values measured using the O_2_ oxygen electrode method (*solid circles*) and from O_2_ exchange (*open circles*) assuming CO_2_ release per oxygenation = 0.5. Shown are the results from *N. tabacum* (**a**), *T. aestivum* (**b**), and *G. max* (**c**).* Bars* represent the means of *n* = 5–7 for the CO_2_ gas exchange data and *n* = 5–16 for the in vitro assays ± *SE*

The temperature response of Γ_*_ was steeper when measured using CO_2_ exchange as compared to that calculated using Rubisco specificity in *N. tabacum, T. aestivum*, and *G. max* (Fig. 2). The differences were most pronounced in *N. tabacum* as compared to *T. aestivum* and *G. max* with the greatest differences being observed at 35 °C, the highest temperature measured. There was a close agreement between the temperature response of Γ_*_ calculated from Rubisco specificity and measurements from O_2_ exchange in *N. tabacum* (Bernacchi et al. 2002, Fig. 2a).

A sensitivity analysis was performed to determine if the differences in Γ_*_ measured using CO_2_ exchange as compared to Γ_*_ measured from Rubisco specificity could be explained by errors in the values of *R*_d_ and *g*_m_ used to convert $C_{\text{i}*}$ to Γ_*_ (see “Materials and methods” section). This sensitivity analysis revealed that in *N. tabacum, G. max*, and *T. aestivum*, the values of *g*_m_ or *R*_d_ would have to be negative to explain the differences between Γ_*_ measured using CO_2_ exchange and Γ_*_ measured from Rubisco specificity at all temperatures, i.e., 25 °C and above (Table 2). Furthermore, *g*_m_ or *R*_d_ would have to be negative or reduced by an order of magnitude to explain the differences in Γ_*_ measured using the two techniques at temperatures below 25 °C. Since the negative values of *g*_m_ and *R*_d_ are not possible, it follows that the temperature-dependent differences in Γ_*_ measured using the two techniques cannot be explained by incorrect assumptions of *g*_m_ or measurements of *R*_d_. Thus, the differences in the values of Γ_*_ measured from CO_2_ exchange vs. in vitro Rubisco specificity are both much too large and in the wrong direction to be explained by errors in *g*_m_ or *R*_d_.

**Fig. 3:**
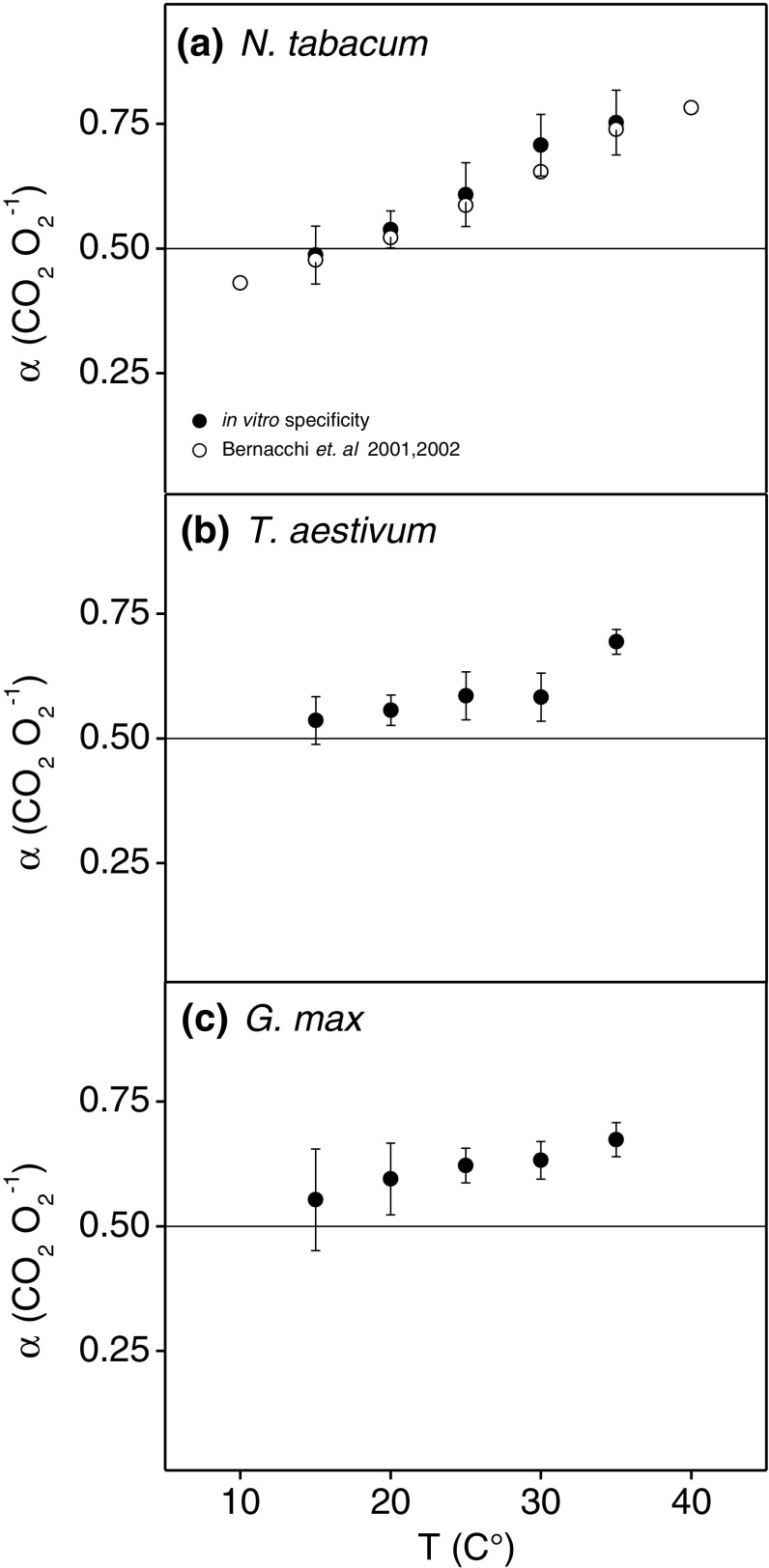
Temperature response of the ratio of CO_2_ release per Rubisco oxygenation (*α*) calculated from photorespiratory CO_2_ compensation points (Γ_*_) measured using the common intersection method and Rubisco specificity values determined using the O_2_ oxygen electrode method (*solid circles*). Also shown are the hypothetical changes in *α* determined from the differences between Γ_*_ measured using CO_2_ and O_2_ exchange in Bernacchi et al. (2001, 2002, *open circles*). Shown are the results from *N. tabacum* (**a**), *T. aestivum* (**b**), and *G. max* (**c**).* Bars* represent the means of *n* = 5–7 for the CO_2_ gas exchange data and *n* = 5–16 for the in vitro assays ± *SE*

Alternatively, increases in *α* with increasing temperature could explain the differences between Γ_*_ observed when measured using CO_2_ exchange, Rubisco specificity, or O_2_ exchange (Fig. 3). The required increase in *α* necessary to explain the difference was largest in *N. tabacum* and consistent when calculated using the values from Rubisco specificity or O_2_ exchange. An increase in *α* of 54% between 15 and 35 °C would be required to explain the difference in Γ_*_ derived by the different determination techniques. Putative increases in *α* were less pronounced, but still large, in *T. aestivum* using the values from Rubisco specificity amounting to a 30% increase in *α* between 15 and 35 °C; however, some of that increase was observed only at 35 °C. When the 35 °C value was removed, the differences in *T. aestivum* and *G. max* were explained by a 22 and 30% increase between 15 and 30 °C, respectively. We next explored how these different Γ_*_ values from the different determination techniques impact higher-scale models of leaf photosynthesis using the values from *N. tabacum*, since these *N. tabacum* parameters are most commonly used to parameterize the FvCB model.

**Fig. 4:**
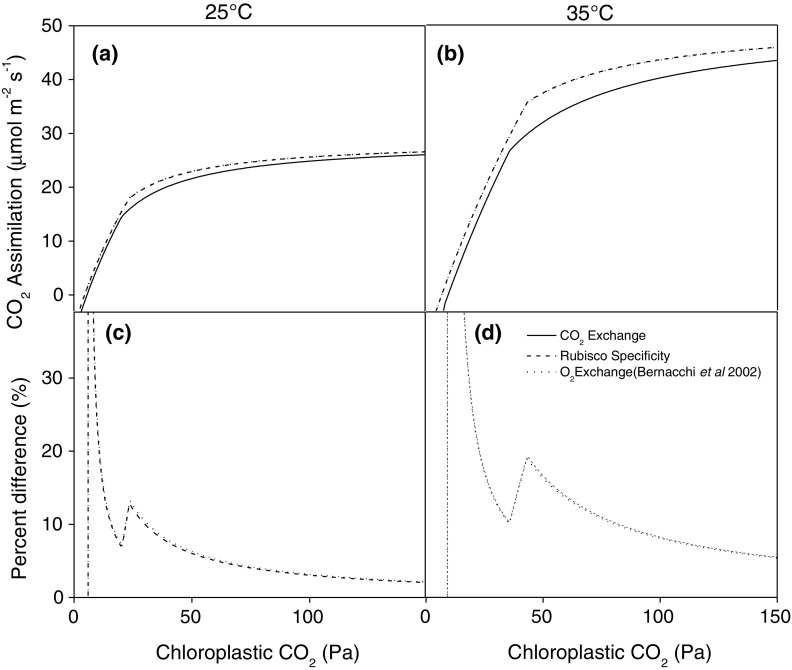
Simulated impact of different assumptions of the photorespiratory CO_2_ compensation point (Γ_*_) on the net CO_2_ assimilation rate at 25 °C (**a, c**) and 35 °C (**b, d**). *Lines* were modeled using the standard biochemical FvCB model of leaf photosynthesis, the temperature response of Rubisco kinetics, the maximum rate of electron transport determined in Bernacchi et al. (2001, 2002), and Γ_*_ assuming the temperature response measured in this study from CO_2_ exchange using the common intersection method (*solid lines*) and from in vitro Rubisco specificity measured using the O_2_ electrode method (*dashed lines*). Shown are the percent differences between net CO_2_ assimilation rate simulated using Γ_*_ measured from CO_2_ exchange and in vitro Rubisco specificity measured using the O_2_ electrode method (*dashed lines*, **c, d**)

Differences in modeled CO_2_ response curves at the leaf level reflected the difference in Γ_*_ values (Fig. 4). The modeled gas exchange using Γ_*_ values measured from CO_2_ exchange were lower than those measured from Rubisco specificity or using O_2_ exchange at 25 and 35 °C by 5 to >40%. The difference increased substantially at 35 °C. The modeled differences were largest at lower CO_2_ partial pressures, where Rubisco kinetics most limit photosynthesis and the model is most sensitive to differences in Rubisco kinetics. The rapid increase in the percent differences at 25 and 35 °C occur during the transition between Rubisco and RuBP regeneration-limited photosynthesis.

**Fig. 5:**
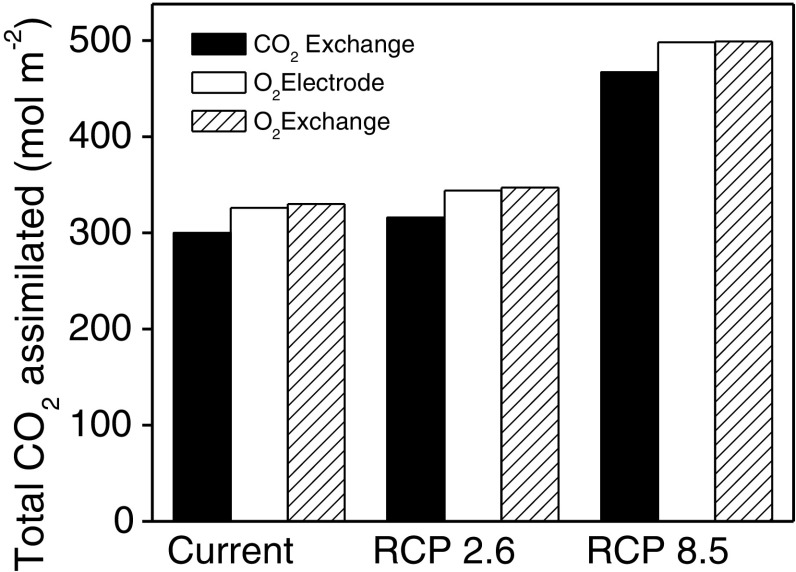
Simulated impact of using different photorespiratory CO_2_ compensation point (Γ_*_) temperature response functions on canopy-level photosynthesis. A multilayer root–canopy model was parameterized with field data from 2002–2005 Bondville, Illinois AmeriFlux eddy covariance experiment assuming the current atmospheric CO_2_ and temperature (400 PPM, no change to air temperature as measured in Bondville), IPCC scenario RCP 2.6 (450 PPM, +1 °C), and IPCC scenario RCP 8.5 (1000 PPM, +3.7 °C). Shown are the total simulated net moles of CO_2_ fixed during the three modeled growing seasons

To understand how using these different temperature responses of Γ_*_ would impact the larger-scale models parameterized with field conditions, we next incorporated each method’s temperature response into a well-validated multilayer canopy model of soybean (MLCan, Drewry et al. 2010a, b, Fig. 5). Since the impact of different Γ_*_ functions are influenced by temperature and CO_2_ concentrations, we ran the model using field data modified according to the current and future climate predictions from the IPCC (Table 1) to produce a realistic range of the present and future conditions. Consistent with the CO_2_ response curve modeling, simulations using Γ_*_ from Rubisco specificity and O_2_ exchange simulate higher net assimilation rates under all conditions. Under the current and RCP 2.6 conditions, simulations using Γ_*_ from Rubisco specificity and O_2_ exchange were 9% greater. Under RCP 8.5 the differences were 7% greater.

**Table 1:** Current and future representative concentration pathways (RCP) of mean global CO_2_ and temperatures according to the 2014 IPCC report

Scenario	Ambient CO_2_ (ppm)	Temp. increase (°C)
Current	400	0.0
100 years RCP 2.6	450	1.0
100 years RCP 8.5	1000	3.7

## Discussion

The differences among Γ_*_ values measured using the different methods revealed an apparent inconsistent temperature response in a critical parameter of photosynthesis that impacts leaf- and canopy-scale simulations of carbon assimilation. Measurements of Γ_*_ derived from CO_2_ gas exchange were the most sensitive to physiological temperature ranges (Fig. 2), and these differences were large enough to result in lower simulated photosynthetic rates as compared to the Γ_*_ values determined from Rubisco specificity or O_2_ exchange. Simulated photosynthesis was especially lower in leaf-level simulations using CO_2_ exchange-based Γ_*_ values under decreased CO_2_ partial pressures at 35 °C (Fig. 4). The differences among methods resulted in more modest differences in photosynthesis when simulated under field conditions at the current and future predictions of climate (Fig. 5). Since intercellular CO_2_ partial pressure is reduced following stomatal closure, the differences in simulated photosynthesis would be greater under stress conditions including drought (Farquhar and Sharkey 1982). These simulations illustrate the sensitivity of the model to parameter values and the importance of understanding why different measurement techniques produce such different Γ_*_ values as the temperature increases.

Γ_*_ measured using CO_2_ exchange does not require the assumption of *α*  = 0.5 that is made in both in vitro and oxygen exchange measurements. This difference can explain the increases in *α* with temperature relative to the other two methods. In *N. tabacum*, the increases in *α* required to harmonize the three methods were similar, indicating that photorespiration may release more carbon than the theoretical minimum as the temperature increases (Fig. 3a). If so, this increased CO_2_ release would decrease the efficiency of photorespiratory recycling of glycolate under elevated temperatures even as the relative rates of Rubisco oxygenation increased due to decreased Rubisco specificity (Badger and Collatz 1978; Jordan and Ogren 1984; Walker et al. 2016b). An increase in *α* could arise through non-enzymatic decarboxylation reactions in the peroxisome of photorespiratory intermediates such as glyoxylate and/or hydroxypyruvate previously suggested to explain all of the photorespiratory CO_2_ loss (Zelitch 1972; Halliwell and Butt 1974; Grodzinski 1978, 1979). This theory was later discounted by numerous lines of genetic and physiological evidence, but only at optimal temperatures (Ogren 1984). Alternatively, excess CO_2_ could be released enzymatically, for example during the generation of carbon skeletons through starch degradation in a proposed glucose 6-phosphate shunt around the Calvin–Benson cycle or an as yet undescribed reaction(s) (Sharkey and Weise 2016). A recent series of isotopic labeling and fluxomic experiments on detached leaves support a stoichiometry of 0.5 in *Helianthus* annuus L. at 21 °C (Abadie et al. 2016), but this value has not yet been confirmed under elevated temperatures or in additional species. Interestingly, the trend in the calculated increases in *α* were not as pronounced in *T. aestivum* or *G. max* (Fig. 3b, c), indicating a potential improvement in photorespiratory efficiency with temperature through selective breeding for yield in these species compared to *N. tabacum*.

Measurements of Γ_*_ from CO_2_ gas exchange require the assumptions of the transfer conductance between the intercellular airspace and the chloroplast (*g*_m_) to accurately calculate chloroplastic CO_2_ concentrations, unlike the measurements of Rubisco specificity or O_2_ exchange (Badger 1985; von Caemmerer 2000; Furbank et al. 2009, Eq. 2). There are several methods for measuring *g*_m_ such as through curve-fitting of CO_2_ response curves, combined gas exchange and chlorophyll fluorescence, and carbon isotope discrimination (Evans et al. 1986; Loreto et al. 1992; Tazoe et al. 2011). While many of these methods have been used to measure the temperature response of *N. tabacum* with similar results, they do show variation, especially at temperatures above 35 °C (Bernacchi et al. 2002; Evans and von Caemmerer 2012; Walker et al. 2013). Given this uncertainty, is it possible that the differences among Γ_*_ measuring techniques result from erroneous assumptions of *g*_m_?

It does not seem probable that errors in the assumptions of *g*_m_, or *R*_d_ for that matter, can explain the differences in Γ_*_ for several reasons. First, Γ_*_ values from CO_2_ exchange were *higher* than those calculated from Rubisco specificity as the temperature increased (Fig. 2), even though Γ_*_ *decreases* with the inclusion of *g*_m_ in the calculation (Eq. 2). This means that *g*_m_ or *R*_d_ would need to *decrease* with temperature to explain the direction of the differences among Γ_*_ measurements, which has not been observed in any reported temperature responses (Bernacchi et al. 2002; Warren and Dreyer 2006; Walker et al. 2013; von Caemmerer and Evans 2014). Furthermore, even if *g*_m_ were assumed to be a negligible value, Γ_*_ in *N. tabacum* at 35 °C would only be reduced from 7.8 to 7.6 Pa, which is insufficient to approach the value of 5.2 Pa determined from Rubisco specificity. This point is further illustrated in the sensitivity analysis of Γ_*_ calculations, where the impossibility of negative values of *g*_m_ or *R*_d_ are required to explain the differences between Γ_*_ measured from CO_2_ exchange and in vitro Rubisco specificity (Table 2). Together, these observations and calculations indicate that the differences between methods of measuring Γ_*_ are not the result of incorrect assumptions or measurements of *R*_d_ and/or *g*_m_.

**Table 2:** Intercellular CO_2_ partial pressure of the common intersection measurements ($C_{\text{i}*}$; Pa CO_2_), the corresponding rates of day respiration (*R*_d_; µmol CO_2_ m^−2^ s^−1^), the assumed mesophyll conductance (*g*_m_; mol m^−2^ s^−1^ MPa^−1^), and the final CO_2_ photocompensation point (Γ_*_; Pa CO_2_) calculated from $C_{\text{i}*}$, *R*_d_, and *g*_m_

*T*_l_	$C_{\text{i}*}$	*R*_d_	${R_{{\text{d}},{S_{{\text{C/O}}}}}}$	*g*_m_	${g_{{\text{m}},{S_{{\text{C}}/{\text{O}}}}}}$	Γ_*_	Γ_*_ $_{{S_{{\text{C/O}}}}}$
*N. tabacum*							
15	2.58 ± 0.14	0.55 ± 0.14	0.09	3.32	0.54	2.75 ± 0.32	2.87 ± 0.02
20	3.33 ± 0.12	1.19 ± 0.07	0.01	4.41	0.05	3.60 ± 0.26	3.38 ± 0.07
25	4.27 ± 0.25	1.34 ± 0.10	−0.10	5.69	−0.42	4.51 ± 0.57	3.70 ± 0.06
30	6.15 ± 0.35	2.08 ± 0.22	−0.23	7.11	−0.78	6.44 ± 0.79	4.53 ± 0.08
35	7.59 ± 0.45	2.32 ± 0.24	−0.27	9.01	−1.04	7.85 ± 1.01	5.18 ± 0.03
*G. max*							
15	2.92 ± 0.27	0.05 ± 0.07	−0.10	2.63	−5.17	2.94 ± 0.60	2.66 ± 0.02
20	3.30 ± 0.23	0.59 ± 0.07	−0.09	4.09	−0.63	3.44 ± 0.50	2.93 ± 0.04
25	4.27 ± 0.14	0.89 ± 0.15	−0.14	4.85	−0.77	4.45 ± 0.31	3.58 ± 0.02
30	5.04 ± 0.17	1.17 ± 0.24	−0.17	5.40	−0.78	5.26 ± 0.39	4.13 ± 0.04
35	6.21 ± 0.20	1.70 ± 0.12	−0.23	6.35	−0.86	6.48 ± 0.44	4.75 ± 0.02
*T. aestivum*							
15	2.59 ± 0.12	0.63 ± 0.16	0.02	3.21	0.09	2.79 ± 0.27	2.65 ± 0.02
20	3.21 ± 0.09	0.70 ± 0.13	−0.04	3.32	−0.18	3.42 ± 0.20	3.09 ± 0.07
25	3.95 ± 0.18	0.88 ± 0.20	−0.10	3.94	−0.43	4.17 ± 0.40	3.57 ± 0.04
30	4.67 ± 0.20	1.17 ± 0.17	−0.11	4.01	−0.37	4.96 ± 0.46	4.23 ± 0.11
35	6.08 ± 0.14	1.88 ± 0.15	−0.38	3.76	−0.77	6.58 ± 0.32	4.64 ± 0.01

Also shown are the Γ_*_ value calculated from in vitro Rubisco specificity (Γ_*_ $_{{S_{{\text{C/O}}}}}$; Pa CO_2_), the *R*_d_ value necessary to explain the differences between $C_{\text{i}*}$ and Γ_*_ $_{{S_{{\text{C/O}}}}}$ (${R_{{\text{d}},{S_{{\text{C/O}}}}}}$), and the g_m_ value necessary to explain the differences between $C_{\text{i}*}$ and Γ_*_ $_{{S_{{\text{C/O}}}}}$ (${g_{{\text{m}},{S_{{\text{C}}/{\text{O}}}}}}$), all according to Eqs. 1 and 2. All data are shown for leaf temperatures (*T*_l_; °C) between 15 and 35 °C. The *g*_m_ values were determined according to the temperature responses measured previously for these species (von Caemmerer and Evans 2014). Shown are the means of *n* = 5–7 for the CO_2_ gas exchange data and *n* = 5–16 for the in vitro assays ± *SE*

Recent work concerning the validity of assumptions necessary for Γ_*_ measurements using the common intersection approach raise important considerations to ensure accurate determinations of Γ_*_. One such concern is the appropriateness of using linear fits to determine the intersection point of non-linear *A*–*C*_i_ curves, which, according to simulations, results in underestimates of $C_{\text{i}*}$ and, by extension, Γ_*_ (Gu and Sun 2014). To prevent assumptins of linearity from baising our common intercept determinations, we used measurement CO_2_ partial pressures below 10 Pa CO_2_ that further simulations demonstrate result in <1% underestimation of $C_{\text{i}*}$ (Walker and Ort 2015). Additionally, the common intersection measurements did not show the “staggered” interceptions expected if their determination was biased by improper assumptions of linearity (Supplemental 1a–c). Finally, even if improper assumptions of linearity resulted in underestimates of $C_{\text{i}*}$ measured in the temperature response of *N. tabacum, G. max*, and *T. aestivum*, this would only serve to *increase* the differences between the Γ_*_ values measured using the common intersection method and those from in vitro Rubisco specificity.

It has also been suggested that the current understanding of *g*_m_ needs to be revised, since it is commonly assumed that all CO_2_ released from the mitochondria passes through the chloroplast, and multiple conductances of CO_2_ between organelles and cytosol need to be considered (Tholen and Zhu 2011; Tholen et al. 2012). A new method of interpreting Γ_*_ measurements from CO_2_ exchange indicates that the relationship between the slope and intercepts of a common intersection measurement would be non-linear in the presence of multiple inter-organellar fluxes from photorespired CO_2_ (Walker and Ort 2015; Walker et al. 2016a). Non-linearity in the slope and intercept relationships was not observed under our growth and measurement conditions, suggesting that an assumption of a simple linear *g*_m_ was justified in this case (Table 2).

An alternative intriguing possibility is that the assumptions used to derive *C*_i_ are not always appropriate and result in a systematic error in the estimation of Γ_*_ in common intersection measurements. This argument rests on the assumption of the FvCB model that the vast majority of water loss occurs through the stomata and through the same path as CO_2_ diffusion (Moss and Rawlins 1963). This assumption has recently been challenged after an analysis of its impact on gas exchange measurements, especially the ones sensitive to small fluxes as in Γ_*_ determination (Hanson et al. 2016). This re-evaluation of a common assumption is supported by the work demonstrating that water diffuses 20–40 times faster across the cuticle than CO_2_ (Boyer et al. 1997; Boyer 2015b) and that many leaves transmit significant amounts of water through the cuticle, resulting in an over-estimation of stomatal conductance and consequently *C*_i_, especially at lower rates of leaf water loss (Boyer 2015a). The impact of cuticular water loss on *C*_i_ estimation would be complex and require additional specialized measurements to determine if these effects could explain the differences observed using CO_2_ gas exchange to measure Γ_*_. Despite the added complexity, the possibility remains that cuticular water loss could explain the differences observed between the Γ_*_ values determined using CO_2_ exchange and those determined based on in vitro Rubisco specificity.

There are two primary methods used to determine the in vitro Rubisco specificity. These alternatively monitor O_2_ consumption via oxygenation of RuBP in an O_2_ electrode system (Parry et al. 1989), or determine the ratio of ^3^H-glycerate/^3^H-glycolate produced from the consumption of ^3^H-RuBP (Kane et al. 1994). While the absolute values produced do differ, there is consistency across methods as to the comparisons across species (e.g., both methods maintain that wheat Rubisco has a higher specificity than *N. tabacum* at 25 °C). Both methods have been employed in model species and a number of crop species under standard temperatures, and datasets incorporating temperature response are available for both methods (e.g., Galmés et al. 2005; Perdomo et al. 2015; Hermida-Carrera et al. 2016; Orr et al. 2016; Prins et al. 2016; Sharwood et al. 2016). However, the difference between methods has not been directly compared with temperature responses, due to a slight overlap of species with temperature response data using both methods. Recent efforts to compile and normalize in vitro Rubisco catalysis data (including *S*_C/O_) from the available literature suggest that the methods available largely agree on the extent of temperature response once in vitro data were calculated accounting for the variation in equilibrium CO_2_ concentration and the ionic strength of buffers (Galmés et al. 2016). This observation suggests that our findings should be relatively consistent with those from the other in vitro methods. The close agreement between Γ_*_ values determined from O_2_ exchange and using Rubisco specificity determined using the O_2_ electrode is remarkable. Clearly, if in vitro specificities are to be used in the modeling efforts of CO_2_ exchange, the method used to collect them should be reported and carefully considered.

In this report, we demonstrate that there are significant differences in the temperature response of Γ_*_ dependent on the measurement method used and that these differences are large enough to impact leaf and canopy models of photosynthesis. While we have limited our discussion to the impact of these different Γ_*_ values to net CO_2_ uptake, similar analysis could be performed to determine the impact to the measurements of *g*_m_ or carbon isotope exchange (Farquhar et al. 1989; Harley et al. 1992; Tholen et al. 2012; Gu and Sun 2014). Given the growing use of biochemical models of leaf photosynthesis to calculate carbon balance and productivity at all scales, it is critical to next reveal the mechanism for these differences in order to determine which methods should be used to accurately parameterize future work or explore novel physiology. The intent of this work is thus not to invalidate the measurements of Γ_*_ using the common intersection method, but rather to determine if more complete physiology can be learned by carefully comparing the Γ_*_ values measured using different techniques. Additionally, the source of these differences could provide insight into the efficiency of photorespiration in response to temperature or the biochemistry of Rubisco.

## Materials and methods

### Plant growth conditions

Plant material used for in vitro measurements was grown in a glasshouse at Rothamsted Research with a 16/8 h day/night cycle and accompanying diurnal temperatures of 26/19 °C. Plants were kept well watered. Young healthy leaves were collected, snap frozen immediately in liquid nitrogen, and then stored at −80 °C until analysis. For CO_2_ gas exchange determination of Γ_*_ at the University of Illinois, *N. tabacum, T. aestivum*, and *G. max* seeds were grown in 2-L pots for 3–5 weeks until large enough for gas exchange. Plants were grown in a climate-controlled cabinet (Conviron, Winnipeg, Manitoba, Canada) set to mimic conditions in the Rothamsted glasshouse with day/night cycles of 16/8 h at 26/19 °C under an irradiance of 800 µmol m^−2^ s^−1^.

### In vitro Rubisco specificity measurements

Rubisco was purified from each species using the material grown in glasshouse conditions at Rothamsted Research, using the method described by Prins et al. (2016), and with alterations as in Orr et al. (2016). The oxygen electrode method of Parry et al. (1989) was used to make a minimum of 12 replicate measurements of *S*_C/O_ for each species, at 15 and 35 °C, and normalized to a known value for *T. aestivum* at each temperature, as described previously (Parry et al. 1989). For 20, 25, and 30 °C, the values from Orr et al. (2016) were used.

### Γ_*_ and *R*_d_ measurements using the common intersection method

The youngest fully expanded leaves of 3- to 5-week-old plants were used for gas exchange. Gas exchange was performed using a LI-COR 6400 XT modified to reach low CO_2_ partial pressures (LI-COR Biosciences 2010) using a 6 cm^2^ chamber with a red/blue light source (LI-COR Biosciences, Lincoln, NE, USA). Assimilation measurements were corrected for CO_2_ leakage according to the manufacturer’s instruction. Γ_*_ was measured using the common intersection method by measuring the CO_2_ response of photosynthesis under various sub-saturating irradiances (Laisk 1977; Brooks and Farquhar 1985). The common intersection was determined using slope–intercept regression to produce more accurate and consistent values of $C_{\text{i}*}$ and *R*_d_ (Walker and Ort 2015; Walker et al. 2016a). To determine irradiances that would result in an even distribution of photosynthetic rates for Γ_*_ determinations, the photosynthetic light response of each species was first measured at 20 Pa CO_2_. Prior to Γ_*_ determinations using the common intersection method, plants were acclimated under 250 µmol m^−2^ s^−1^ at 39 Pa CO_2_ until photosynthesis reached steady state to activate Rubisco. Following initial acclimation, plants were measured at 15, 12, 9, 7, 5, and 3 Pa CO_2_ under irradiances of 250, 165, 120, 80, and 50 µmol m^−2^ s^−1^ for *N. tabacum*, 250, 160, 100, 60, and 30 µmol m^−2^ s^−1^ for *T. aestivum*, and 250, 165, 120, 80, and 50 µmol m^−2^ s^−1^ for *G. max*. The x-intersection point represents $C_{\text{i}*}$ which can be converted to Γ_*_ according to

2 $${{{\varGamma }}_*} = C_{{{\text{i}*}}} + {R_{\text{d}}}/{g_{\text{m}}},$$

where *R*_d_ is the y-intersection point (von Caemmerer 2000; Furbank et al. 2009). Species-specific temperature responses were used at each temperature for *g*_m_ (von Caemmerer and Evans 2014).

### Leaf- and canopy-scale modeling of photosynthesis

Leaf-level modeling of the CO_2_ response of net photosynthesis was modeled at 25 and 35 °C using the standard FvCB model of leaf photosynthesis. For 25 °C, the model was parameterized with *V*_cmax_ = 80 µmol m^−2^ s^−1^, *K*_c_ = 26.7 Pa, *K*_o_ = 16.3 kPa, *R*_d_ = 1 µmol m^−2^ s^−1^, and *J*_max_ = 120 µmol m^−2^ s^−1^. Γ_*_ was assumed to be 4.74, 3.78, and 3.7 Pa for the common intersection method, O_2_ exchange, and in vitro determinations, respectively. For 35 °C, the model was parameterized with *V*_cmax_ = 187 µmol m^−2^ s^−1^, *K*_c_ = 77.1 Pa, *K*_o_ = 22.2 kPa, *R*_d_ = 2 µmol m^−2^ s^−1^, and *J*_max_ = 211 µmol m^−2^ s^−1^, respectively. Γ_*_ was assumed to be 7.88, 5.15, and 5.2 Pa for the common intersection method, O_2_ exchange, and in vitro determinations, respectively.

For canopy-level implementation, we used a well-validated multilayer canopy–root–soil model (MLCan, Drewry et al. 2010a, b) with minor additions to include *g*_m_ (Walker et al. 2016b). The model was parameterized with field data from the Bondville, Illinois, AmeriFlux eddy covariance site measured during the 2002, 2004, and 2006 growing seasons (available from the AmeriFlux Database; http://ameriflux.lbl.gov/data/download-data). Full-field data can also be obtained from B. J. W. upon request.

## Electronic supplementary material

Below is the link to the electronic supplementary material.

Supplementary material 1 (DOCX 633 KB)

## References

[CR1] Abadie C, Boex-Fontvieille ERA, Carroll AJ, Tcherkez G (2016). In vivo stoichiometry of photorespiratory metabolism. Nat Plants.

[CR2] Badger MR (1985). Photosynthetic oxygen exchange. Ann Rev Plant Physiol.

[CR3] Badger MR, Collatz GJ (1978). ) Studies on the kinetic mechanism of ribulose-1,5-bisphosphate carboxylase and oxygenase reactions, with particular reference to the effect of temperature on kinetic parameters. Carnegie Inst Wash Yearb.

[CR4] Bernacchi CJ, Singsaas EL, Pimentel C, Portis AR, Long SP (2001). Improved temperature response functions for models of Rubisco-limited photosynthesis. Plant Cell Environ.

[CR5] Bernacchi CJ, Portis AR, Nakano H, von Caemmerer S, Long SP (2002). Temperature response of mesophyll conductance. Implications for the determination of Rubisco enzyme kinetics and for limitations to photosynthesis in vivo. Plant Physiol.

[CR6] Boyer JS (2015). Impact of cuticle on calculations of the CO_2_ concentration inside leaves. Planta.

[CR7] Boyer JS (2015). Turgor and the transport of CO_2_ and water across the cuticle (epidermis) of leaves. J Exp Bot.

[CR8] Boyer JS, Wong SC, Farquhar GD (1997). CO_2_ and water vapor exchange across leaf cuticle (epidermis) at various water potentials. Plant Physiol.

[CR9] Brooks A, Farquhar GD (1985). Effect of temperature on the CO_2_/O_2_ specificity of ribulose-1,5-bisphosphate carboxylase/oxygenase and the rate of respiration in the light. Planta.

[CR10] Drewry DT, Kumar P, Long S, Bernacchi C, Liang XZ, Sivapalan M (2010). Ecohydrological responses of dense canopies to environmental variability: 1. Interplay between vertical structure and photosynthetic pathway. J Geophys Res.

[CR11] Drewry DT, Kumar P, Long S, Bernacchi C, Liang XZ, Sivapalan MCG (2010). Ecohydrological responses of dense canopies to environmental variability: 2. Role of acclimation under elevated CO_2_. J Geophys Res.

[CR12] Dufresne J-L, Foujols M-A, Denvil S, Caubel A, Marti O, Aumont O, Balkanski Y, Bekki S, Bellenger H, Benshila R, Bony S, Bopp L, Braconnot P, Brockmann P, Cadule P, Cheruy F, Codron F, Cozic A, Cugnet D, de Noblet N, Duvel J-P, Ethé C, Fairhead L, Fichefet T, Flavoni S, Friedlingstein P, Grandpeix J-Y, Guez L, Guilyardi E, Hauglustaine D, Hourdin F, Idelkadi A, Ghattas J, Joussaume S, Kageyama M, Krinner G, Labetoulle S, Lahellec A, Lefebvre M-P, Lefevre F, Levy C, Li ZX, Lloyd J, Lott F, Madec G, Mancip M, Marchand M, Masson S, Meurdesoif Y, Mignot J, Musat I, Parouty S, Polcher J, Rio C, Schulz M, Swingedouw D, Szopa S, Talandier C, Terray P, Viovy N, Vuichard N (2013). Climate change projections using the IPSL-CM5 Earth System Model: from CMIP3 to CMIP5. Clim Dynam.

[CR13] Evans JR, von Caemmerer S (2012). Temperature response of carbon isotope discrimination and mesophyll conductance in tobacco. Plant Cell Environ.

[CR14] Evans J, Sharkey T, Berry J, Farquhar G (1986). Carbon isotope discrimination measured concurrently with gas exchange to investigate CO_2_ diffusion in leaves of higher plants. Funct Plant Biol.

[CR15] Farquhar GD, Sharkey TD (1982). Stomatal conductance and photosynthesis. Ann Rev Plant Physiol.

[CR16] Farquhar GD, von Caemmerer S, Berry JA (1980). A biochemical model of photosynthetic CO_2_ assimilation in leaves of C_3_ species. Planta.

[CR17] Farquhar GD, Ehleringer JR, Hubick KT (1989). Carbon isotope discrimination and photosynthesis. Ann Rev Plant Physiol Plant Mol Biol.

[CR18] Furbank RT, von Caemmerer S, Sheehy J, Edwards GE (2009). C_4_ rice: a challenge for plant phenomics. Funct Plant Biol.

[CR19] Galmés J, Flexas J, Keys AJ, Cifre J, Mitchell RAC, Madgwick PJ, Haslam RP, Medrano H, Parry MAJ (2005). Rubisco specificity factor tends to be larger in plant species from drier habitats and in species with persistent leaves. Plant Cell Environ.

[CR20] Galmés J, Hermida-Carrera C, Laanisto L, Niinemets Ü (2016). A compendium of temperature responses of Rubisco kinetic traits: variability among and within photosynthetic groups and impacts on photosynthesis modeling. J Exp Bot.

[CR21] Grodzinski B (1978). Glyoxylate decarboxylation during photorespiration. Planta.

[CR22] Grodzinski B (1979). A study of formate production and oxidation in leaf peroxisomes during photorespiration. Plant Physiol.

[CR23] Gu L, Sun Y (2014). Artefactual responses of mesophyll conductance to CO_2_ and irradiance estimated with the variable *J* and online isotope discrimination methods. Plant Cell Environ.

[CR24] Halliwell B, Butt VS (1974). Oxidative decarboxylation of glycolate and glyoxylate by leaf peroxisomes. Biochem J.

[CR25] Hanson DT, Stutz SS, Boyer JS (2016). Why small fluxes matter: the case and approaches for improving measurements of photosynthesis and (photo) respiration. J Exp Bot.

[CR26] Harley P, Weber J, Gates D (1985). Interactive effects of light, leaf temperature, CO_2_ and O_2_ on photosynthesis in soybean. Planta.

[CR27] Harley P, Loreto F, Di Marco G, Sharkey T (1992). Theoretical considerations when estimating the mesophyll conductance to CO_2_ flux by analysis of the response of photosynthesis to CO_2_. Plant Physiol.

[CR28] Hermida-Carrera C, Kapralov MV, Galmés J (2016). Rubisco catalytic properties and temperature response in crops. Plant Physiol.

[CR29] Jordan DB, Ogren WL (1984). The CO_2_/O_2_ specificity of ribulose 1,5-bisphosphate carboxylase/oxygenase. Planta.

[CR30] Kane H, Viil J, Entsch B, Paul K, Morell M, Andrews T (1994). An improved method for measuring the CO_2_/O_2_ specificity of ribulosebisphosphate carboxylase-oxygenase. Funct Plant Biol.

[CR31] Kromdijk J, Long SP (2016) One crop breeding cycle from starvation? How engineering crop photosynthesis for rising CO_2_ and temperature could be one important route to alleviation. Proc Royal Soc B. doi:10.1098/rspb.2015.2578 10.1098/rspb.2015.2578 PMC4810849 26962136

[CR32] Laisk A (1977). Kinetics of photosynthesis and photorespiration in C_3_ plants.

[CR33] LI-COR Biosciences (2010) Modification of LI-6400/LI-6400XT to control at low [CO_2_]. LI-COR application note Application note 7

[CR34] Long SP, Marshall-Colon A, Zhu X-G (2015). Meeting the global food demand of the future by engineering crop photosynthesis and yield potential. Cell.

[CR35] Loreto F, Harley PC, Di Marco G, Sharkey TD (1992). Estimation of mesophyll conductance to CO_2_ flux by three different methods. Plant Physiol.

[CR36] Moss DN, Rawlins SL (1963). Concentration of carbon dioxide inside leaves. Nature.

[CR37] Ogren WL (1984). Photorespiration: pathways, regulation, and modification. Ann Rev Plant Physiol.

[CR38] Orr D, Alcântara A, Kapralov MV, Andralojc J, Carmo-Silva E, Parry MAJ (2016). Surveying Rubisco diversity and temperature response to improve crop photosynthetic efficiency. Plant Physiol.

[CR39] Parry MAJ, Keys AJ, Gutteridge S (1989). Variation in the specificity factor of C3 higher plant Rubiscos determined by the total consumption of Ribulose-P2. J Exp Bot.

[CR40] Perdomo JA, Cavanagh AP, Kubien DS, Galmés J (2015). Temperature dependence of in vitro Rubisco kinetics in species of *Flaveria* with different photosynthetic mechanisms. Photosyn Res.

[CR41] Prins A, Orr DJ, Andralojc PJ, Reynolds MP, Carmo-Silva E, Parry MAJ (2016). Rubisco catalytic properties of wild and domesticated relatives provide scope for improving wheat photosynthesis. J Exp Bot.

[CR42] Ruuska SA, Badger MR, Andrews TJ, von Caemmerer S (2000). Photosynthetic electron sinks in transgenic tobacco with reduced amounts of Rubisco: little evidence for significant Mehler reaction. J Exp Bot.

[CR43] Sharkey TD, Weise SE (2016). The glucose 6-phosphate shunt around the Calvin–Benson cycle. J Exp Bot.

[CR44] Sharwood RE, Ghannoum O, Kapralov MV, Gunn LH, Whitney SM (2016). Temperature responses of Rubisco from Paniceae grasses provide opportunities for improving C3 photosynthesis. Nat Plants.

[CR45] Tazoe Y, von Caemmerer S, Estavillo GM, Evans JR (2011). Using tunable diode laser spectroscopy to measure carbon isotope discrimination and mesophyll conductance to CO_2_ diffusion dynamically at different CO_2_ concentrations. Plant Cell Environ.

[CR46] Tholen D, Zhu X-G (2011). The mechanistic basis of internal conductance: a theoretical analysis of mesophyll cell photosynthesis and CO_2_ diffusion. Plant Physiol.

[CR47] Tholen D, Ethier G, Genty B, Pepin S, Zhu X-G (2012). Variable mesophyll conductance revisited: theoretical background and experimental implications. Plant Cell Environ.

[CR48] von Caemmerer S (2000). Biochemical models of leaf photosynthesis.

[CR49] von Caemmerer S, Evans JR (2014). Temperature responses of mesophyll conductance differ greatly between species. Plant Cell Environ.

[CR50] von Caemmerer S, Farquhar GD (1981). Some relationships between the biochemistry of photosynthesis and the gas exchange of leaves. Planta.

[CR51] Walker BJ, Cousins A (2013). Influence of temperature on measurements of the CO_2_ compensation point: differences between the Laisk and O_2_-exchange methods. J Exp Bot.

[CR52] Walker BJ, Ort DR (2015). Improved method for measuring the apparent CO_2_ photocompensation point resolves the impact of multiple internal conductances to CO_2_ to net gas exchange. Plant Cell Environ.

[CR53] Walker BJ, Ariza LS, Kaines S, Badger MR, Cousins AB (2013). Temperature response of in vivo Rubisco kinetics and mesophyll conductance in *Arabidopsis thaliana*: comparisons to *Nicotiana tabacum*. Plant Cell Environ.

[CR54] Walker BJ, Skabelund DC, Busch FA, Ort DR (2016). An improved approach for measuring the impact of multiple CO_2_ conductances on the apparent photorespiratory CO_2_ compensation point through slope-intercept regression. Plant Cell Environ.

[CR55] Walker BJ, VanLoocke A, Bernacchi CJ, Ort DR (2016). The costs of photorespiration to food production now and in the future. Ann Rev Plant Biol.

[CR56] Warren C, Dreyer E (2006). Temperature response of photosynthesis and internal conductance to CO_2_: results from two independent approaches. J Exp Bot.

[CR57] Zelitch I (1972). The photooxidation of glyoxylate by envelope-free spinach chloroplasts and its relation to photorespiration. Arch Biochem Biophys.

[CR58] Zhu XG, Portis AR, Long SP (2004). Would transformation of C3 crop plants with foreign Rubisco increase productivity? A computational analysis extrapolating from kinetic properties to canopy photosynthesis. Plant Cell Environ.

[CR59] Zhu X-G, Long SP, Ort DR (2008). What is the maximum efficiency with which photosynthesis can convert solar energy into biomass?. Curr Opin Biotechnol.

