# Supplementary material for: Uncertainty in measurements of the photorespiratory CO2 compensation point and its impact on models of leaf photosynthesis
Source: Photosynth Res. 2017 Mar 28;132(3):245–55. doi: 10.1007/s11120-017-0369-8 (PMC5443873; doi:10.1007/s11120-017-0369-8)
Supplement: Supplementary file 1 — Supplementary material 1 (DOCX 633 KB) [file 11120_2017_369_MOESM1_ESM.docx]

**Supplemental 1a:** Common intersection measurements for *Nicotiana tabacum* at 15-35 °C. Shown are means of n=5-7 ± SE.

**Supplemental 1b:** Common intersection measurements for *Glycine max* at 15-35 °C. Shown are means of n=5-7 ± SE.

**Supplemental 1c:** Common intersection measurements for *Triticum aestivum* at 15-35 °C. Shown are means of n=5-7 ± SE.

**Supplemental 2:** Residual plot of slope-intercept regressions derived from common intersection measurements in *Nicotiana tabacum*. Shown are the differences between the measured slope-intercept coordinate and a linear regression used to determine C_i*_ and R_d_ values.
